# Identification of B Cell Epitopes of Alcohol Dehydrogenase Allergen of *Curvularia lunata*

**DOI:** 10.1371/journal.pone.0020020

**Published:** 2011-05-25

**Authors:** Smitha Nair, Neetu Kukreja, Bhanu Pratap Singh, Naveen Arora

**Affiliations:** 1 Allergy and Immunology Laboratory, Institute of Genomics and Integrative Biology (CSIR), Delhi, India; 2 Department of Zoology, Hindu College, Delhi University, Delhi, India; University Hospital Freiburg, Germany

## Abstract

**Background/Objective:**

Epitope identification assists in developing molecules for clinical applications and is useful in defining molecular features of allergens for understanding structure/function relationship. The present study was aimed to identify the B cell epitopes of alcohol dehydrogenase (ADH) allergen from *Curvularia lunata* using *in-silico* methods and immunoassay.

**Method:**

B cell epitopes of ADH were predicted by sequence and structure based methods and protein-protein interaction tools while T cell epitopes by inhibitory concentration and binding score methods. The epitopes were superimposed on a three dimensional model of ADH generated by homology modeling and analyzed for antigenic characteristics. Peptides corresponding to predicted epitopes were synthesized and immunoreactivity assessed by ELISA using individual and pooled patients' sera.

**Result:**

The homology model showed GroES like catalytic domain joined to Rossmann superfamily domain by an alpha helix. Stereochemical quality was confirmed by Procheck which showed 90% residues in most favorable region of Ramachandran plot while Errat gave a quality score of 92.733%. Six B cell (P1–P6) and four T cell (P7–P10) epitopes were predicted by a combination of methods. Peptide P2 (epitope P2) showed E(X)_2_GGP(X)_3_KKI conserved pattern among allergens of pathogenesis related family. It was predicted as high affinity binder based on electronegativity and low hydrophobicity. The computational methods employed were validated using Bet v 1 and Der p 2 allergens where 67% and 60% of the epitope residues were predicted correctly. Among B cell epitopes, Peptide P2 showed maximum IgE binding with individual and pooled patients' sera (mean OD 0.604±0.059 and 0.506±0.0035, respectively) followed by P1, P4 and P3 epitopes. All T cell epitopes showed lower IgE binding.

**Conclusion:**

Four B cell epitopes of *C. lunata* ADH were identified. Peptide P2 can serve as a potential candidate for diagnosis of allergic diseases.

## Introduction

Allergic diseases have multi-factorial aetiology induced by interplay of allergens (usually proteins) and immune system components. The allergens derived from pollens, insects, animals and fungi have been implicated in allergy. They trigger activation of B and T cells, cross-linking of effector cell bound IgE and release of inflammatory mediators inducing allergic response [1]. Epitopes, the molecular determinants present on allergens are involved in determining the type and intensity of the immune response. Identification of IgE binding and activation of CD4+ helper T cells in response to recognition of allergen epitopes on MHC class II molecules play a crucial role in development of the immune response [2].

Fungal allergens have been recognized as a leading cause of allergic asthma [3]. The incidence of mold allergy ranges from 6 to 24% in the general population, up to 44% among atopics and 80% among asthmatics [4]. The mold *C. lunata* has been implicated as an important causative agent in allergic rhinitis, asthma and allergic bronchopulmonary mycoses [5]. Aerobiological studies have reported a prevalence of 2–10% of *C. lunata* spores at different places in India, North America and other Asian countries [6], [7]. *C. lunata* sensitizes 7–16% of atopic population in India and 18–28% in other countries [8]. The mold harbours several cross reactive and unique allergens [5]. Previously, cytochrome c, enolase and serine protease were identified as major allergens of *C. lunata* [8]–[10]. Further, a 38 kDa polypeptide was identified as an allergen of *C. lunata* by screening cDNA library with allergic patients' sera. The molecule was identified as alcohol dehydrogenase (ADH) based on its sequence homology (unpublished data). Alcohol dehydrogenases are glycolytic enzymes involved in NAD^+^ regeneration. However, the role of fungal ADHs as allergens is largely unexplored. Previous studies have described a 40 kDa ADH as the major allergen of *Candida albicans* [11].

Epitopes have been elucidated by enzymatic fragmentation, synthetic overlapping peptides, site directed mutagenesis, pepscan assay, screening of random phage display libraries, recombinant peptide libraries and X- ray crystallography [12]–[15]. Computational algorithms offer fairly accurate and rapid determination of epitope location on the allergen molecule [1], [16]. The present study was aimed to determine the molecular structure of *C. lunata* ADH and to identify the B and T cell epitopes following *in-silico* approaches. An integrated strategy was employed for B cell epitope prediction by combining results from sequence and structure based methods and protein-protein interaction tools. Qualitative and quantitative methods were used for prediction of promiscuous peptides binding to HLA class II molecules. Synthetic peptides representing the predicted epitopes were evaluated by ELISA using *C. lunata* positive patients' sera.

## Materials and Methods

### Sequence

Alcohol dehydrogenase was cloned from *C. lunata* and sequenced in the laboratory (NCBI accession no. **ABC88428**).

### Template Selection

The *C. lunata* ADH (CADH) sequence was searched for homology in the Protein Data Bank (PDB). The homologous template suitable for ADH was selected by BLAST server (http://ncbi.nlm.nih.gov/). The BioInfoBank Metaserver (http://meta.bioinfo.pl/) which employs fold recognition for homology search was also used for template selection. The crystal structure of template was retrieved from PDB and used for homology modeling.

### Sequence Alignment

Amino acid sequence of target (CADH) and template protein was aligned using Clustal W multiple sequence alignment tool (http://www.ebi.ac.uk/Tools/clustalw/) with default parameters. The output was manually checked to optimize the alignment. The alignment data was used for homology modeling of CADH.

### Secondary Structure Prediction

The secondary structure was predicted using PHD (http://npsa-pbil.ibcp.fr/cgi-bin/npsa_automat.pl?page=/NPSA/npsa_phd.html) and PSIPRED (http://bioinf.cs.ucl.ac.uk/psipred/) fold servers.

### Allergenicity prediction

The allergenicity of CADH was predicted by a full FASTA alignment in the Structural Database of Allergenic Proteins (SDAP) and Allermatch allergen database.

### Homology Modeling

The template was modeled based on the crystal structure of *Saccharomyces cerevisiae* ADH (SADH) using the academic version of MODELLER9v4 [17]. Couple of residues at the N-terminus of CADH could not be modeled due to lack of homology with the template. Several models were generated and evaluated using GA341 and Discrete Optimized Protein Energy (DOPE) assessment functions of MODELLER. Stereochemical quality of the selected model was evaluated using PROCHECK (http://nihserver.mbi.ucla.edu/SAVES_3/), PROSA (https://prosa.services.came.sbg.ac.at/) Errat (http://nihserver.mbi.ucla.edu/) and WHATCHECK (http://nihserver.mbi.ucla.edu/SAVES_3/saves.php) programs. The incorrectly modeled loop regions were identified and refined using MODELLER. The final model was optimized by steepest descent algorithm of the Amber force field implemented in the SYBIL software from Tripos Inc. (St. Louis, MO). The stereochemical quality of the model was evaluated once again by the above mentioned tools. The CADH model was superimposed on the template crystal structure to calculate average distance between their Cα backbones. The visualization of the model was accomplished by Jmol (http://www.jmol.org/) and Chimera softwares (http://www.cgl.ucsf.edu/chimera).

### Prediction of B cell epitopes

For determination of linear epitopes, the sequence of ADH was submitted to ABCpred, BCPREDS, Bcepred, Antigenic, BepiPred 1.0b and Ellipro web servers [18]–[26]. Amino acid propensity scales of Hopp -Woods (hydrophilicity), Emini (Surface probability), Jameson-Wolf (antigenic index) and Karplus-Schulz (flexibility) were used as implemented in the Protean application of DNASTAR Lasergene version 7.2. (DNASTAR Inc., Madison, WI, USA) For prediction of discontinuous epitopes, the structure generated by homology modeling was submitted to Discotope 1.2 server and conformational epitope prediction server (CEP) [27], [28]. Protein-protein interaction sites on the 3D model of *C. lunata* ADH were predicted using cons PPISP and PPI-PRED online tools [29], [30]. The methods were selected on the basis of performance measures that include both threshold dependent (sensitivity, specificity, accuracy) and independent parameters. While selecting consensus epitopes, a preference was given to results predicted from tools tested on larger datasets and having an area under receiver operating characteristic curve (AUC) greater than 0.65. Tools based solely on physico-chemical parameters were given the least preference. Default settings were applied to all the tools used. The regions recognized frequently by four or more than four tools were selected.

### Prediction of T cell epitopes

HLA class II binding peptides of CADH were predicted by inhibitory concentration (IC_50_) value based quantitative prediction methods MHCpred SVRMHC and SMM-Align [31]–[33]. Online tools Propred, Multipred, SVMHC, and RANKPEP which employ binding status scoring qualitative prediction methods were also used for predicting T cell epitopes [34]–[37]. Promiscuous peptides binding to multiple HLA class II molecules were selected.

### Solvent accessibility and electrostatic potential

Solvent accessibility of all the residues in the generated model was calculated by *ASAView* (http://www.netasa.org/asaview/). Absolute surface area (ASA) of each residue was calculated by DSSP program. These values were transformed to relative values of ASA. Electrostatic potential for the protein was calculated by Protein continuum electrostatics (PCE) tool (http://bioserv.rpbs.jussieu.fr/cgi-bin/PCE-Pot) based on the MEAD (macroscopic electrostatics with atomic detail) program and employing parse parameters. Electrostatic energies are generated via finite difference solution to the Poisson–Boltzmann equation. Protein and surrounding solvent are assigned a dielectric constant of 4.0 and 80.0, respectively. Ionic strength value is maintained at 0.1.

B and T cell epitopes recognised by computational tools were mapped onto linear sequence and on the three dimensional model of CADH to determine their position and secondary structure.

Patients' Sample: Patients of asthma, rhinitis or both were screened based on history, skin tests with allergen extracts, clinical and laboratory investigations at Out Patients' Department, V.P. Chest Institute, Delhi. Asthma was diagnosed as per American Thoracic Society guidelines [38] and rhinitis was assigned to patients(s) having two or more of the symptoms recommended in ARIA [39]. Blood samples were collected from 12 *Curvularia* hypersensitive patients and sera were used for immunoassay.

### *C. lunata* native extract and recombinant ADH

*C. lunata* was cultivated, fungal mat was harvested and spore-mycelial extract was prepared as described elsewhere [6]. ADH was cloned in pET22b+ expression vector and expressed in *E. coli* cells as a recombinant protein (unpublished data).

### Peptide synthesis

The ten predicted epitopes (peptides) were synthesized by a manufacturer (GL Biochem, Shanghai, China) with a purity of ≥90% and their sequences were confirmed by mass spectrometry (MALDITOF) analyses.

### Enzyme linked immunosorbent assay (ELISA)

The microtiter plates (Covalink Nunc _TM_ Immunomodule, Roskilde, Denmark) were coated with 1 µg/100 µl/well of *Curvularia* native extract, 250 ng/100 µl/well of recombinant ADH or 100 ng/100 µl/well of synthetic peptides in carbonate-bicarbonate buffer (pH 9.6). The plates were blocked with 3% defatted milk (200 µl/well) for 3 h at 37°C. After washing the plate with PBST (0.1 M Phosphate buffer saline with 0.05% Tween 20), individual patients' sera (1∶10 v/v) or pooled patients' sera (1∶10 v/v) from *Curvularia* positive patients were added and the plate incubated overnight at 4°C. The IgE binding was probed with anti-human IgE horseradish peroxidase (1∶1000 v/v; Sigma, USA). The colour was developed with orthophenylenediamine and hydrogen peroxide in citrate buffer. The absorbance was read at 492 nm on an ELISA reader (Benchmark Plus, Bio-Rad) after stopping the reaction with 5 N sulphuric acid. Sera from 5 healthy subjects were used as control.

## Results

### Template selection

Among the proteins with known tertiary structure in the Protein Data Bank (PDB), *Saccharomyces cerevisiae* alcohol dehydrogenase (SADH I, pdb accession number: 2HCY_A) showed highest sequence identity of 55% and sequence similarity of 72% with CADH. The suitability of selected model was checked by BioInfoBank Metaserver which returned a 3D Jury score (Jscore) of 279.71. A significant 3D-Jury score (>50) correlated with the quality of the prediction. The crystal structure of SADH was retrieved from PDB and used for homology modeling.

### Sequence alignment

The primary sequence of CADH showed presence of all 22 residues that have been proposed to be conserved among ADH molecules across various kingdoms [40] and of these, 50% are glycine residues (Figure S1). The region from 67–82 amino acids of CADH represents the zinc binding motif associated with alcohol dehydrogenases. The region 179–185 shows GAGGGLG sequence, which correlates with the pattern of GXGXXG that occurs in the coenzyme (NAD) binding domain of dehydrogenases. The N-terminal mitochondrial targeting sequence is absent suggesting that it may be a cytoplasmic protein.

### Secondary structure prediction

Secondary structure prediction with PHD server identified 9 α helices and 16 β sheets in CADH whereas PSIPRED server predicted 12 α helices and 15 β sheets. The alpha helical regions recognized by PSIPRED and PHD were superimposed on 3-D structure of CADH. The α helices, β pleated sheets and coils predicted by PSIPRED correlated well with the modeled 3-D structure.

### Allergenicity prediction

Sequence of CADH showed an identity of 59.66% with *Candida albicans* ADH in SDAP server and 59.59% in Allermatch server.

### Generation of models based on template

A total of 400 structures simulating SADH were generated using MODELLER 9v4. Best structure model was selected based on DOPE potential and GA341 score. Since all the models returned a GA341 score of 1, the model with the lowest DOPE score (−38736.0625) was selected. Model quality was assessed using the programs PROCHECK, PROSA and Errat. Three loops corresponding to the regions 60–69, 79–86 and 204–206 exceeded >99% error values in Errat. These regions were optimized using loop refinement function of MODELLER. Fifty independent conformations were modeled and the best resulting model with the lowest DOPE score was selected. The refined model was subjected to energy minimization using the steepest descent algorithm implemented in the Sybil software. The resulting model was once again checked for stereochemical quality with the above mentioned tools.

### Quality of model

The overall stereochemical parameters as evaluated by online tools are consistent with a model of 2.0 Å resolution.

#### a) Procheck

**Figure 1:**
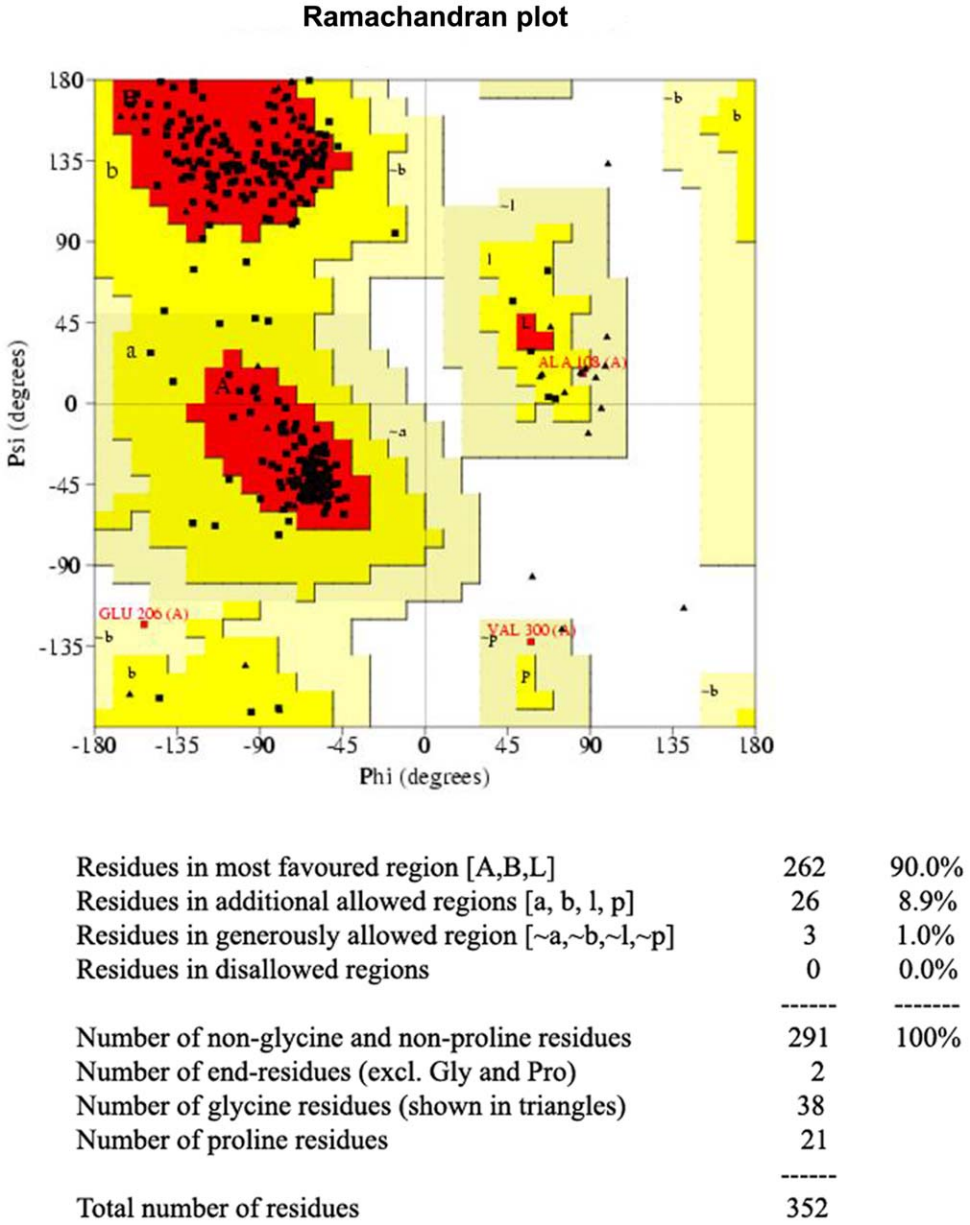
Ramachandran plot for *Curvularia* alcohol dehydrogenase. Ramachandran plot shows the amino acid residues in the *C. lunata* alcohol dehydrogenase model. In CADH 90% of residues were present in most favourable region, 8.9% in additional allowed region and 1% in generously allowed region. No residues were detected in the disallowed region.

The main chain conformations of modeled protein were located in the acceptable regions of the Ramachandran plot. Majority of residues (90%) were in most favourable regions of the ϕ and ψ values, 8.9% of the residues in the allowed regions whereas 1% of the residues were in generously allowed regions. No residues were present in the disallowed regions (Figure 1). The plot of χ_1_ versus χ_2_ torsion angles for each residue shows that most of the rotamers in CADH are localized in low energy regions. Five residues have their χ_2_ angle in a high energy zone.

#### b) ProSa

A Z score of −8.42 was returned in ProSa. The score was within the range of scores usually found for native proteins of similar size.

#### c) ERRAT

**Figure 2:**
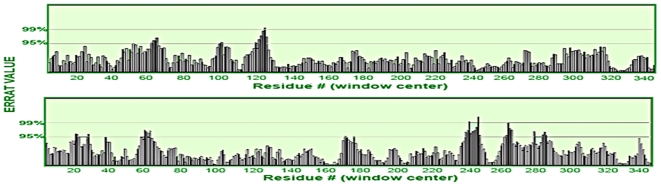
ERRAT plot for CADH structure. Error values for residues as predicted by ERRAT for CADH structure. Y axis presents the error value and X axis presents the amino acid sequences of SADH (top) and CADH (bottom), respectively. An error value exceeding 99% confidence level indicates poorly modelled regions. The overall quality factor assigned to template and CADH structures are 94.232% and 92.733%, respectively.

An overall quality factor of 94.232% was assigned by Errat for SADH structure while a quality factor of 92.733% was assigned to CADH structure (Figure 2).

#### d) WHAT_CHECK defines the refinement constraint values in the form of rms z score (Table 1), which is a measure of deviation from the expected behaviour for different parameters

**Table 1:** Summary of CADH model properties.

Property	CADH model
Sequence identity	55%
Sequence similarity	72%
Resolution of template structure	2.4 Å
Backbone RMSD from template	0.4 Å
rms Z-score for bond lengths	0.520
rms Z-score for bond angles	1.172
rms Z-score for ω angle restraints	1.490
rms Z-score for side chain planarity	1.788
rms Z-score for improper dihedral distribution	1.027
rms Z-score for inside/outside distribution	1.023
Chi 1/chi 2 correlation Z-score	−0.954
Residues with Z-score for torsion angles <−2	5
Final energy (force field)	−8637.853 KJ/mol
Final electrostatic constraints energy	−5116.52 KJ/mol

Values close to 1 are considered ideal.

**Figure 3:**
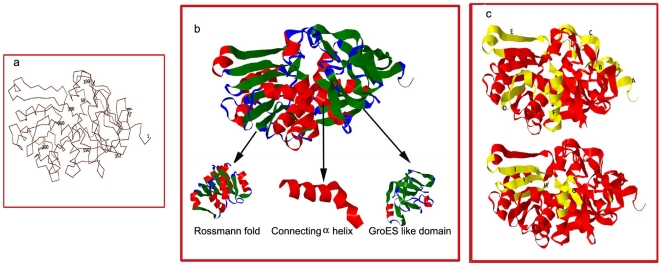
Structure of CADH. (a) The alpha carbon backbone of *C. lunata* alcohol dehydrogenase is numbered from 1–352. (b) Structure of *C. lunata* alcohol dehydrogenase derived from homology modelling could be distinguished into three regions namely Rossmann fold, connecting helix and a GroES like domain. (c) The predicted B and T cell epitopes were localized on the tertiary structure of CADH. The top panel shows six B cell epitopes whereas bottom panel shows four T cell epitopes. The epitopes are depicted in yellow colour on the red ribbon structure. The molecular graphics images were produced using Jmol.

The α carbon structural backbone of CADH is presented in figure 3a. The homology modeled structure of CADH showed two independent domains joined by a long alpha helix (Figure 3b). The N-terminal region (32–141 amino acids) is the catalytic domain of ADH and has a GroES like structure. It appears as an irregular β barrel composed of 4 strands with a partly open roof hairpin. The region 171–315 has NAD binding Rossmann superfamily domain consisting of 2 symmetrical β-α-β-α-β supersecondary units. This region exhibits an outer layer of α helices packed against a central core of parallel β sheets. Each Rossmann fold binds one nucleotide moiety of the NAD (cofactor) molecule. The GroES like domain of ADH is connected to the Rossmann fold by a long α helix spanning 20 amino acid residues (146–166). Root mean square deviation (RMSD) between CADH and template Cα backbone was 0.4 Å.

### B-cell epitope prediction

Six antigenic regions were predicted most common in CADH by several prediction tools used in the present study. These six regions are 1: 1–9, 2: 14–34, 3: 51–68, 4: 164–184, 5: 276–293 and 6: 299–316 (Table 2). All the B cell epitopes were located on the surface of the protein molecule. Glycine (14.28%) followed by proline (10.47%) and lysine (9.52%) were most common residues in epitopes. Hydrophobic content was analysed to identify regions with a higher probability of interaction with immunoglobulin. Around 60% of the 105 residues were non polar, 23.8% were charged residues and 16.19% were recorded as polar residues for the predicted epitopes. The relative surface accessibility values calculated by *ASAView* showed 52.38% residues with greater than 30% whereas 33.33% residues were with greater than 50% solvent accessibility.

**Table 2:** Predicted B and T cell epitopes of *C. lunata* ADH.

Peptide	Sequence^a^	Type of epitope	Position	Tools^b^	IgE binding (OD_492_)^c^
P1	MsnIPq**E**qW	B	1–9	5,6,8,9	0.463±0.0055
P2	**EK**tGGPV**E**y**KK**IPVq**K**PGP**DE**	B	14–34	1,2,4,5,6,7,8,9	0.506±0.0035
P3	AVnG**D**WPLPt**K**LPLVGG**H**	B	51–68	1,2,4,5,6,8,9,10	0.405±0.0005
P4	L**KE**sGV**K**PGqFAAIVGAGGGL	B	164–184	2,4,5,6,7,9,11	0.429±0.0045
P5	PAGAyL**K**APVF**E**tVI**K**MI	B	276–293	1,3,4,6,10,11	0.133±0.0065
P6	yVGn**RKD**ss**E**AI**E**FF**RR**G	B	299–316	1,2,5,7,8	0.078±0.003
P7	VnI**K**FsGVC**H**t	T	37–47	12,13,15,16,17	0.197±0.001
P8	VVILVAVn**EK**	T	246–255	12,13,15,16,17	0.253±0.0055
P9	yV**R**P**R**GtVIC	T	263–272	12,13,15,16,17	0.271±0.017
P10	VI**K**MI**R**IqGsyVGn**RKD**s	T	289–307	13,14,15,16,17,18	0.206±0.006

a Non polar residues are shown in upper case, polar residues in lower case and charged residues are depicted in bold.

b Tools that recognised the corresponding B or T cell epitope or a part of the epitope are numbered as follows: (1) ABCpred, (2) BCPREDS, (3) Bcepred, (4) Antigenic, (5) BepiPred 1.0b, (6) Ellipro, (7) DNASTAR, (8) Discotope server, (9) CEP server, (10) cons PPISP, (11) PPI-PRED, (12) MHCpred, (13) SVRMHC, (14) SMM-Align, (15) Propred, (16) Multipred, (17) SVMHC, (18) RANKPEP.

c For IgE binding, peptides were tested by ELISA using *Curvularia* positive patients' sera. The control serum samples were drawn from healthy individuals. Control value IgE = 0.122±0.01 (mean ± SE). The specific IgE values represented here are mean values of duplicates.

### T cell epitope prediction

Seven publicly available tools trained on different datasets were employed for determination of HLA class II binders. Four amino acid sequences (7: 37–47, 8: 246–255, 9: 263–272 and 10: 289–307) were identified as promiscuous binders by comparison of prediction data. The predicted epitopes comprised of 50 residues with a high frequency of occurrence for valine (20%) and isoleucine (12%). Half (50%) of the total residues were non polar whereas 28% were polar. Charged residues (22%) were least preferred in the T cell epitopes. Only 32% and 18% of residues in T cell epitopes had solvent accessibility greater than 30% and 50%, respectively. All four T cell epitopes are primarily composed of β sheets. The epitopes P7, P8 and P9 are localized in the interior of the protein molecule whereas epitope P10 is solvent accessible and shows an overlap of 8 residues with a B cell epitope.

### Electrostatic potential

**Figure 4:**
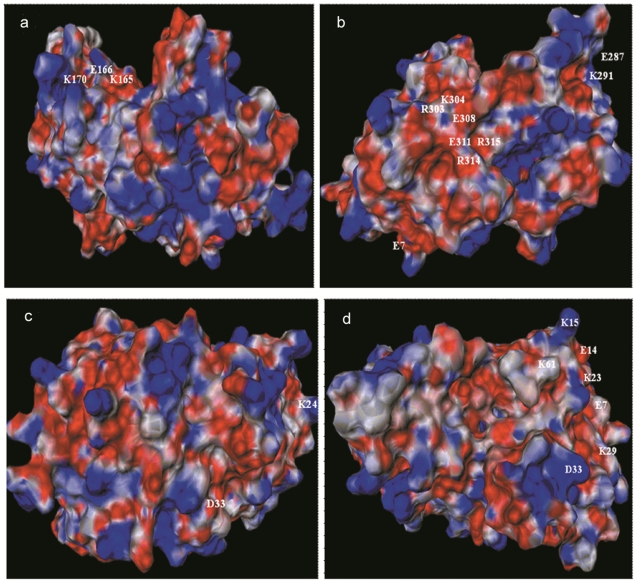
Electrostatic potential of CADH. Blue colour represents values more than 2 kcal/mol/e. Values between 0 to 2 kcal/mol/e is shown as blue gradient. Red colour represents values less than −2 kcal/mol/e. Red gradient shows electrostatic potential between −2 to 0 kcal/mol/e. The charged residues falling in the predicted epitopes are labelled. a) image centered on “auto”. b) Y rotation by 90° c) X rotation by 90° d) X+Y rotations by 90°.

The B and T cell epitopes were located on the 3D structure designed (Figure 3c). The surface of CADH model displays several prominent charged residues with half of the side exhibiting large positive values (Figure 4a, blue regions) and the other half showing predominantly negative values (Figure 4b, red regions). By assigning a value of +1 to basic residues (His, Arg, Lys) and −1 to the acidic residues (Asp, Glu), net charge of protein was calculated to be +8.

### ELISA

**Figure 5:**
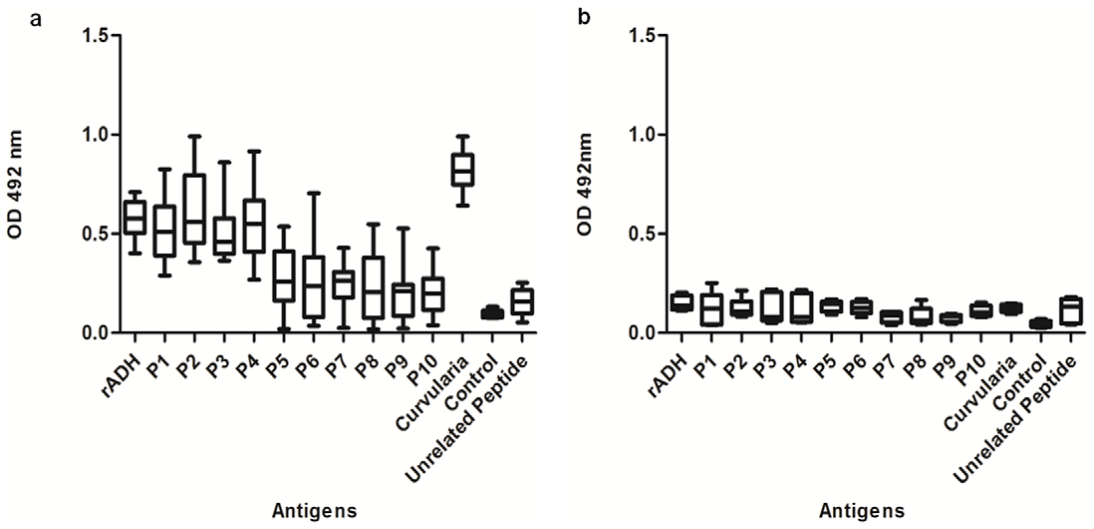
IgE binding studies. (a) B and T cell peptide binding was tested with 12 individual *Curvularia* hypersensitive patients' sera. *Curvularia* native, recombinant ADH or peptides were coated on ELISA plate and incubated with hypersensitive patients' sera. The IgE affinity was detected by HRP labelled anti human IgE. (b) *Curvularia* native, recombinant ADH or peptides were coated on ELISA plate, probed with sera from 5 healthy subjects and detected by HRP labelled anti human IgE. The data is presented as box and whiskers plot showing variation of IgE values from median. All values show p<0.05 as compared to control.

Synthetic peptides representing the epitopes were tested for IgE binding by ELISA using individual patients' sera or pooled sera from *Curvularia* hypersensitive patients (Figures 5a and 5b, Table 2). The cut off value for ELISA positive for individual patients' sera was taken as 3 times of the control NHS (OD 0.095±0.005). IgE binding against an unrelated peptide was also checked to determine non-specific binding, if any. Peptide P2 (epitope P2) showed maximum IgE binding with individual and pooled patients' sera (mean OD 0.604±0.059 and 0.506±0.0035, respectively). Peptides P1, P3 and P4 showed significantly higher IgE binding (p<0.05) as compared to control (OD 0.095±0.005) whereas peptides P5–P10 demonstrated lower IgE binding. Peptides P1–P3 showed high IgE binding with 12/12 of sera whereas P4 showed IgE binding with 11/12 of sera. Peptides P5 and P6 showed high IgE binding with 5/12 and 6/12 of patients' sera, respectively. Individual sera from healthy volunteers (n = 5) showed low IgE binding in the range of OD 0.074–0.15 with *Curvularia*, recombinant ADH and peptides in comparison to patients' sera.

## Discussion

Interactions among B and T cells play an important role in the aetiology of allergic response. The specific stimulants (epitopes) of such interactions are of paramount importance in determining the immune response. Numerous databases of experimentally derived epitopes have aided in development of algorithms for predicting epitopes of hitherto uncharacterized proteins. The quality of these computational tools for epitope prediction of proteins has been investigated [2], [41]. The algorithm TEPITOPE was used to predict promiscuous HLA-DR ligands of Lol p 5a allergen from rye grass [1]. Sharma et al. predicted five B and five T cell epitopes of major allergen Cur l 3 from *C. lunata* which correlates with experimental data [16]. The predicted B cell epitopes showed a higher IgE binding than T cell epitopes whereas T cell epitopes demonstrated higher PBMCs proliferation compared to B cell epitopes. In the present study, potential IgE and HLA Class II binding regions of CADH were defined by *in silico* methods and validated experimentally.

ADHs exist in dimeric or tetrameric form [40] and SADH is a tetramer comprising of four identical subunits. However, it is yet to be ascertained that *C. lunata* ADH exists in such multimeric form. In the present study, CADH 3D model was generated not only to provide a framework for localization of predicted epitopes but also to delineate properties of these predicted regions. The model showed permissible bond angles and vaander waal's radii. Slight error was (>95%) recorded for residues 234–238 in ERRAT owing to poor homology. The stereochemical evaluation showed it to be a good model for ADH and different parameters were used to improve the efficiency of prediction.

Oezguen et al. showed that glycine and lysine have a high propensity to lie in IgE binding sites [42]. In addition, studies demonstrated that allergen epitopes comprise a high proportion of hydrophobic amino acids [43], [44]. The most common residues in the B cell epitopes of CADH are glycine followed by proline and lysine. Further, more than half of the total residues lying in B cell epitopes were hydrophobic.

SDAP database search revealed homology of epitope B of CADH with allergens of pathogenesis related (PR-10) family. Epitope P2 shows E(X)_2_GGP(X)_3_KKI pattern, which is conserved in Bet v 1, Car b 1, Aln g 1, Pha v 1, Gly m 4 and Ara h 8. The conformational B cell epitope of major birch pollen allergen includes first 8 residues of this conserved sequence [15]. Hence the pattern found in CADH P2 may be significant in determining its allergenicity.

Electrostatic interactions determine orientation of the molecules and stabilize antigen-antibody complexes. In the present study, both the epitopes P2 and P3 showed a strong negative potential, though scattered blue regions are observed due to lysine residues in P2. Sinha *et al* showed strong electrostatic interactions and lower hydrophobic content promote high affinity Ag-Ab complex formation [45]. Thus epitope P2 with a strong negative potential and low hydrophobicity of 44.44% was predicted as a high affinity binder. This correlates with the ELISA results which show high IgE binding for P2 as compared to control (OD 0.506±0.0035).

IgE binding requires partial accessibility for solvent. Here CADH showed that half of residues have relative solvent accessiblity of 30% or more indicating a possibility for immunoglobulin binding. *ASAView* output showed hydrophobic (proline, valine, leucine and isoleucine) and charged residues (arginine, lysine) on the exterior of the plot implying high solvent accessibility. Brinda and Vishveshwara demonstrated that hydrophobic residues can function as weak interface hubs [46]. Further, solvent accessible arginine may interact with several amino acid residues facilitating subunit interaction. Arginine plays a major role as a central hub by interacting with residues from same subunit and from other subunits.

A crucial step in development of allergic response is activation of CD4+ T cells that recognise antigenic epitopes in conjunction with HLA class II molecules. Hence CADH sequence was investigated for HLA class II binding regions using qualitatitive and quantitative MHC class II binding prediction methods. The presence of a large number of HLA alleles made selection of potential T cell epitopes a daunting task. Hence, promiscuous peptides were selected that bind to multiple HLA class II molecules, which makes them suitable candidates for vaccine development and immunotherapy [37]. Computational tools predicting MHC Class II peptides are however limited in defining T cell recognition of the bound peptides. Also factors such as co-stimulatory molecules are essential for induction of T cell proliferation to the allergen molecule. Hence further experimental work is required to validate these epitopes.

IgE binding studies with predicted peptides show that BepiPred 1.0b, Ellipro and CEP servers have predicted all the four high binders correctly. BCPREDS, antigenic and discotope servers have identified 3 whereas ABCpred and DNASTAR each have predicted one epitope correctly. Thus the structure based servers using spatial information and surface availability/accessibility perform much better as compared to tools based solely on physicochemical properties of antigen/allergen. The use of string kernels or recurrent neural networks combined with physicochemical properties is another interesting possibility for development of tools for epitope identification. Prediction of the binding residue(s) in the epitope and its mutagenesis to reduce allergenicity *in silico* could pave the way for development of novel tools in immunoinformatics.

Computational validation of epitope prediction methods used here was performed with Bet v 1 (birch pollen) and Der p 2 (house dust mite) allergens having experimentally validated epitopes [15], [47]–[50]. The 3D structure and sequence(s) were retrieved from Protein Data Bank and NCBI and both B and T cell epitopes were predicted for these proteins, using computational tools employed in the present study. The computational predictions identified 67% of Bet v 1 and 60% of Der p 2 known epitopes correctly. Apart from this, some new epitopes were also predicted.

In conclusion, the present study identified four B cell epitopes using a combination of bioinformatics tools and IgE binding with patients'sera. The structural and physico-chemical properties of the predicted epitopes are consistent with that of known allergen epitopes. The *in-silico* methods employed here for epitope prediction are validated with known allergenic proteins namely Bet v 1 (birch pollen) and Der p 2 (house dust mite). Similar approach may be employed for prediction of epitopes in novel proteins.

## Supporting Information

Figure S1 **Sequence alignment of CADH with ADH from different sources.** The sequences of alcohol dehydrogenase from various sources were aligned using Clustal W (1.81) multiple sequence alignment tool (http://align.genome.jp/) and manually optimized. The sequences are as follows: 1) Chain A of human alpha alcohol dehydrogenase, 2) Chain A, human beta-1 alcohol dehydrogenase, 3) Alcohol dehydrogenase E chain from *Equus caballus* (horse), 4) alcohol dehydrogenase from *Rattus norvegicus* (Norway rat), 5) Chain A of mouse alcohol dehydrogenase from *Mus musculus*, 6) alcohol dehydrogenase 1 from *Zea mays*, 7) alcohol dehydrogenase from *Arabidopsis thaliana*, 8) alcohol dehydrogenase II from *Candida albicans*, 9) alcohol dehydrogenase from *Candida albicans*, 10) alcohol dehydrogenase from *Saccharomyces cerevisiae*, 11) alcohol dehydrogenase from *Cochliobolous lunatus*, 12) alcohol dehydrogenase from *Schizosaccharomyces pombe*. *Curvularia lunata* ADH (CADH) accession number is highlighted in blue whereas the conserved residues are denoted by an asterisk (*). CADH sequence shows the presence of all 22 residues conserved across kingdoms as reported by Jornvall et al., 1987 (40). These residues are highlighted in yellow.
(DOCX)
