# Supplementary material for: Identification of B Cell Epitopes of Alcohol Dehydrogenase Allergen of Curvularia lunata
Source: PLoS One. 2011 May 25;6(5):e20020. doi: 10.1371/journal.pone.0020020 (PMC3102081; doi:10.1371/journal.pone.0020020)
Supplement: Figure S1 — Sequence alignment of CADH with ADH from different sources. The sequences of alcohol dehydrogenase from various sources were aligned using Clustal W (1.81) multiple sequence alignment tool (http://align.genome.jp/) and manually optimized. The sequences are as follows: 1) Chain A of human alpha alcohol dehydrogenase, 2) Chain A, human beta-1 alcohol dehydrogenase, 3) Alcohol dehydrogenase E chain from Equus caballus (horse), 4) alcohol dehydrogenase from Rattus norvegicus (Norway rat), 5) Chain A of mouse alcohol dehydrogenase from Mus musculus, 6) alcohol dehydrogenase 1 from Zea mays, 7) alcohol dehydrogenase from Arabidopsis thaliana, 8) alcohol dehydrogenase II from Candida albicans, 9) alcohol dehydrogenase from Candida albicans, 10) alcohol dehydrogenase from Saccharomyces cerevisiae, 11) alcohol dehydrogenase from Cochliobolous lunatus, 12) alcohol dehydrogenase from Schizosaccharomyces pombe. Curvularia lunata ADH (CADH) accession number is highlighted in blue whereas the conserved residues are denoted by an asterisk (*). CADH sequence shows the presence of all 22 residues conserved across kingdoms as reported by Jornvall et al., 1987 (40). These residues are highlighted in yellow. (DOCX) [file pone.0020020.s001.docx]

Supporting Information

Figure S1: Sequence alignment of CADH with ADH from different sources

gi|13096739|pdb|1HSO|A_Chain_A ----------STAGKVIKCKAAVLWELKKP--FSIEEVEVAPPKAHEVRI

gi|13096741|pdb|1HSZ|A_Chain_A ----------STAGKVIKCKAAVLWEVKKP--FSIEDVEVAPPKAYEVRI

gi|113397|sp|P00327.2|ADH1E_HO ---------MSTAGKVIKCKAAVLWEEKKP--FSIEEVEVAPPKAHEVRI

gi|202727|gb|AAA40681.1|_alcoh ---------MSTAGKVIKCKAAVLWEPHKP--FTIEDIEVAPPKAHEVRI

gi|10835861|pdb|1E3E|A_Chain_A ----------GTQGKVIKCKAAIAWKTGSP--LCIEEIEVSPPKACEVRI

gi|162460397|ref|NP_001105409. ---------MATAGKVIKCKAAVAWEAGKP--LSIEEVEVAPPQAMEVRV

gi|22136298|gb|AAM91227.1|_alc MENGNSSSDNKSSHKPIRCKAAVSRKAGEP--LVMEEIMVAPPQPFEVRI

gi|3859714|emb|CAA21988.1|_alc ------------MSVPTTQKAVIFETNGGK--LEYKDIPVPKPKANELLI

gi|608690|emb|CAA57342.1|_alco ----------MSEQIPKTQKAVVFDTNGGQ--LVYKDYPVPTPKPNELLI

gi|171021|gb|AAA34408.1|_alcoh ------------MSIPETQKAIIFYESNGK--LEHKDIPVPKPKPNELLI

gi|86278351|gb|ABC88428.1|_alc -----------MSNIPQEQWAQVIEKTGGP--VEYKKIPVQKPGPDEVLV

gi|12643994|sp|P00332.2|ADH_SC ------------MTIPDKQLAAVFHTHGGPENVKFEEVPVAEPGQDEVLV

* : . :. * * *: :

gi|13096739|pdb|1HSO|A_Chain_A KMVAVGICGTDDHVVSGT--MVTPLPVILGHEAAGIVESVGEGVTTVKPG

gi|13096741|pdb|1HSZ|A_Chain_A KMVAVGICRTDDHVVSGN--LVTPLPVILGHEAAGIVESVGEGVTTVKPG

gi|113397|sp|P00327.2|ADH1E_HO KMVATGICRSDDHVVSGT--LVTPLPVIAGHEAAGIVESIGEGVTTVRPG

gi|202727|gb|AAA40681.1|_alcoh KMVATGVCRSDDHAVSGS--LFTPLPAVLGHEGAGIVESIGEGVTCVKPG

gi|10835861|pdb|1E3E|A_Chain_A QVIATCVCPTDINATDPK--KKALFPVVLGHECAGIVESVGPGVTNFKPG

gi|162460397|ref|NP_001105409. KILFTSLCHTDVYFWEAKG-QTPVFPRIFGHEAGGIIESVGEGVTDVAPG

gi|22136298|gb|AAM91227.1|_alc RIICTALCHSDVTFWKLQV-PPACFPRILGHEAIGVVESVGENVKEVVEG

gi|3859714|emb|CAA21988.1|_alc NVKYSGVCHTDLHAWKGDWPLATKLPLVGGHEGAGVVVALGENVKGWKVG

gi|608690|emb|CAA57342.1|_alco HVKYSGVCHTDLHARKGDWPLATKLPLVGGHEGAGVVVGMGENVKGWKIG

gi|171021|gb|AAA34408.1|_alcoh NVKYSGVCHTDLHAWHGDWPLPTKLPLVGGHEGAGVVVGMGENVKGWKIG

gi|86278351|gb|ABC88428.1|_alc NIKFSGVCHTDLHAVNGDWPLPTKLPLVGGHEGAGVVVARGELVNDVELG

gi|12643994|sp|P00332.2|ADH_SC NIKYTGVCHTDLHALQGDWPLPAKMPLIGGHEGAGVVVKVGAGVTRLKIG

.: :* :* . :* : *** *:: * *. *

gi|13096739|pdb|1HSO|A_Chain_A DKV-IPLAIPQCGKCRICKNPESNYCLKN-DVSNP----QGTLQDGTSRF

gi|13096741|pdb|1HSZ|A_Chain_A DKV-IPLFTPQCGKCRVCKNPESNYCLKN-DLGNP----RGTLQDGTRRF

gi|113397|sp|P00327.2|ADH1E_HO DKV-IPLFTPQCGKCRVCKHPEGNFCLKN-DLSMP----RGTMQDGTSRF

gi|202727|gb|AAA40681.1|_alcoh DKV-IPLFSPQCGKCRICKHPESNLCCQTKNLTQP----KGALLDGTSRF

gi|10835861|pdb|1E3E|A_Chain_A DKV-IPFFAPQCKRCKLCLSPLTNLCGKLRNFKYPTI-DQELMEDRTSRF

gi|162460397|ref|NP_001105409. DHV-LPVFTGECKECAHCKSAESNMCDLLRINTDR----GVMIADGKSRF

gi|22136298|gb|AAM91227.1|_alc DTV-LPTFMPDCGDCVDCKSHKSNLCSKFPFKVSP----WMPRYDNSSRF

gi|3859714|emb|CAA21988.1|_alc DYAGVKWLNGSCLNCEYCQSGAEPNCAEADLSGYTHDG------------

gi|608690|emb|CAA57342.1|_alco DFAGIKWLNGSCMSCEFCQQGAEPNCGEADLSGYTHDG------------

gi|171021|gb|AAA34408.1|_alcoh DYAGIKWLNGSCMACEYCELGNESNCPHADLSGYTHDG------------

gi|86278351|gb|ABC88428.1|_alc DHVGVKWLNGSCLSCDYCQTADEPLCPKPLLSGYTVDG------------

gi|12643994|sp|P00332.2|ADH_SC DRVGVKWMNSSCGNCEYCMKAEETICPHIQLSGYTVDG------------

* . : .* * * *

gi|13096739|pdb|1HSO|A_Chain_A TCR-RKPIHHFLGISTFSQYTVVDENAVAKIDAASPLEKVCLIGCGFSTG

gi|13096741|pdb|1HSZ|A_Chain_A TCR-GKPIHHFLGTSTFSQYTVVDENAVAKIDAASPLEKVCLIGCGFSTG

gi|113397|sp|P00327.2|ADH1E_HO TCR-GKPIHHFLGTSTFSQYTVVDEISVAKIDAASPLEKVCLIGCGFSTG

gi|202727|gb|AAA40681.1|_alcoh SCR-GKPIHHFLSTSTFSQYTVVDDIAVAKIDAAAPLDKVCLIGCGFSTG

gi|10835861|pdb|1E3E|A_Chain_A TCK-GRSIYHFMGVSSFSQYTVVSEANLARVDDEANLERVCLIGCGFSSG

gi|162460397|ref|NP_001105409. SIN-GKPIYHFVGTSTFSEYTVMHVGCVAKINPQAPLDKVCVLSCGYSTG

gi|22136298|gb|AAM91227.1|_alc TDLNGETLFHFLNVSSFSEYTVLDVANVVKIDSSIPPSRACLLSCGVSTG

gi|3859714|emb|CAA21988.1|_alc ---------------SFQQYATADAVQAARIPAGTDLANVAPILCAGVTV

gi|608690|emb|CAA57342.1|_alco ---------------SFEQYATADAVQAAKIPAGTDLANVAPILCAGVTV

gi|171021|gb|AAA34408.1|_alcoh ---------------SFQEYATADAVQAAHIPQGTDLAEVAPILCAGITV

gi|86278351|gb|ABC88428.1|_alc ---------------TFQQYCIAKAAHVARIPKECDLAAIAPVLCAGITV

gi|12643994|sp|P00332.2|ADH_SC ---------------TFQHYCIANATHATIIPESVPLEVAAPIMCAGITC

:*..* . : . : *. :

gi|13096739|pdb|1HSO|A_Chain_A YGSAVNVAKVTPGSTCAVFGL-GGVGLSAIMGCKAAGAARIIAVDINKDKF

gi|13096741|pdb|1HSZ|A_Chain_A YGSAVNVAKVTPGSTCAVFGL-GGVGLSAVMGCKAAGAARIIAVDINKDKF

gi|113397|sp|P00327.2|ADH1E_HO YGSAVKVAKVTQGSTCAVFGL-GGVGLSVIMGCKAAGAARIIGVDINKDKF

gi|202727|gb|AAA40681.1|_alcoh YGSAVQVAKVTPGSTCAVFGL-GGVGLSVVIGCKTAGAAKIIAVDINKDKF

gi|10835861|pdb|1E3E|A_Chain_A YGAAINTAKVTPGSTCAVFGL-GCVGLSAIIGCKIAGASRIIAIDINGEKF

gi|162460397|ref|NP_001105409. LGASINVAKPPKGSTVAVFGL-GAVGLAAAEGARIAGASRIIGVDLNPSRF

gi|22136298|gb|AAM91227.1|_alc VGAAWETAKVEKGSTVVIFGL-GSIGLAVAEGARLCGASRIIGVDINPTKF

gi|3859714|emb|CAA21988.1|_alc Y-KALKTAELEAGQWVAISGAAGGLG-SLAVQYAKAMGYRVLAIDGGEDKG

gi|608690|emb|CAA57342.1|_alco Y-KALKTADLAAGQWVAISGAGGGLG-SLAVQYARAMGLRVVAIDGGDEKG

gi|171021|gb|AAA34408.1|_alcoh Y-KALKSANLRAGHWAAISGAAGGLG-SLAVQYAKAMGYRVLGIDGGPGKE

gi|86278351|gb|ABC88428.1|_alc Y-KGLKESGVKPGQFAAIVGAGGGLG-SLACQYAKAMGVRTIAIDAGEEKK

gi|12643994|sp|P00332.2|ADH_SC Y-RALKESKVGPGEWICIPGAGGGLG-HLAVQYAKAMAMRVVAIDTGDDKA

. : : * : * * * . . : :.:* . :

gi|13096739|pdb|1HSO|A_Chain_A AKAKEL-GATECINPQDYKKP-IQEVLKEMTDGGVDFSFEVIGRLDTMMAS

gi|13096741|pdb|1HSZ|A_Chain_A AKAKEL-GATECINPQDYKKP-IQEVLKEMTDGGVDFSFEVIGRLDTMMAS

gi|113397|sp|P00327.2|ADH1E_HO AKAKEV-GATECVNPQDYKKP-IQEVLTEMSNGGVDFSFEVIGRLDTMVTA

gi|202727|gb|AAA40681.1|_alcoh AKAKEL-GATDCINPQDYTKP-IQEVLQEMTDGGVDFSFEVIGRLDTMTSA

gi|10835861|pdb|1E3E|A_Chain_A PKAKAL-GATDCLNPRELDKP-VQDVITELTAGGVDYSLDCAGTAQTLKAA

gi|162460397|ref|NP_001105409. EEARKF-GCTEFVNPKDHNKP-VQEVLAEMTNGGVDRSVECTGNINAMIQA

gi|22136298|gb|AAM91227.1|_alc QVGQKF-GVTEFVNSMTCEKNRVSEVINEMTDGGADYCFECVGSSSLVQEA

gi|3859714|emb|CAA21988.1|_alc -EFVKSLG-AETFIDFTKEKDVVEAVKKATN-G-GPHGVINVSVSERAIGQ

gi|608690|emb|CAA57342.1|_alco -EFVKSLG-AEAYVDFTKDKDIVEAVKKATD-G-GPHGAINVSVSEKAIDQ

gi|171021|gb|AAA34408.1|_alcoh -ELFTSLG-GEVFIDFTKEKDIVSAVVKATN-G-GAHGIINVSVSEAAIEA

gi|86278351|gb|ABC88428.1|_alc KMCVNDLG-AETFVDFSTSKNLVADVQKATPDGLGPHVVILVAVNEKPFQQ

gi|12643994|sp|P00332.2|ADH_SC -ELVKSFG-AEVFLDFKKEADMIEAVKAATN-G-GAHGTLVLSTSPKSYEQ

* : : * * .

gi|13096739|pdb|1HSO|A_Chain_A LLCCHEACGTSVIVGVPPDSQNLSMNPMLLLTG-RTWKGAILGGFKSKEC

gi|13096741|pdb|1HSZ|A_Chain_A LLCCHEACGTSVIVGVPPASQNLSINPMLLLTG-RTWKGAVYGGFKSKEG

gi|113397|sp|P00327.2|ADH1E_HO LSCCQEAYGVSVIVGVPPDSQNLSMNPMLLLSG-RTWKGAIFGGFKSKDS

gi|202727|gb|AAA40681.1|_alcoh LLSCHSACGVSVIVGVPPSAQSLSVNPMSLLLG-RTWKGAIFGGFKSKDA

gi|10835861|pdb|1E3E|A_Chain_A VDCTVLGWGSCTVVGAKVDEMTIPT--VDVILG-RSINGTFFGGWKSVDS

gi|162460397|ref|NP_001105409. FECVHDGWGVAVLVGVPHKDAEFKTHPMNFLNE-RTLKGTFFGNYKPRTD

gi|22136298|gb|AAM91227.1|_alc YACCRQGWGKTITLGVDKPGSQICLDSFDVLHHGKILMGSLFGGLKAKTH

gi|3859714|emb|CAA21988.1|_alc STEYVRTLGKVVLVGLP-AGAKISTPVFDAVIKTIQIKGSYVGNRKDTAE

gi|608690|emb|CAA57342.1|_alco SVEYVRPLGKVVLVGLP-AHAKVTAPVFDAVVKSIEIKGSYVGNRKDTAE

gi|171021|gb|AAA34408.1|_alcoh STRYCRANGTVVLVGLP-AGAKCSSDVFNHVVKSISIVGSYVGNRADTRE

gi|86278351|gb|ABC88428.1|_alc AAEYVRPRGTVICIGLP-AGAYLKAPVFETVIKMIRIQGSYVGNRKDSSE

gi|12643994|sp|P00332.2|ADH_SC AAGFARPGSTMVTVSMP-AGAKLGADIFWLTVKMLKICGSHVGNRIDSIE

. :. . *: *.

gi|13096739|pdb|1HSO|A_Chain_A VPKLVADFMAKKFSLDALITHVLPFEKINEGFDLLHSGKSIRTILMF---

gi|13096741|pdb|1HSZ|A_Chain_A IPKLVADFMAKKFSLDALITHVLPFEKINEGFDLLHSGKSIRTVLTF---

gi|113397|sp|P00327.2|ADH1E_HO VPKLVADFMAKKFALDPLITHVLPFEKINEGFDLLRSGESIRTILTF---

gi|202727|gb|AAA40681.1|_alcoh VPKLVADFMAKKFPLEPLITHVLPFEKINEAFDLLRAGKSIRTVLTF---

gi|10835861|pdb|1E3E|A_Chain_A VPNLVSDYKNKKFDLDLLVTHALPFESINDAIDLMKEGKSIRTILTF---

gi|162460397|ref|NP_001105409. LPNVVELYMKKELEVEKFITHSVPFAEINKAFDLMAKGEGIRCIIRMEN-

gi|22136298|gb|AAM91227.1|_alc IPILLKRYLSNELELDKFVTHEMKFEEINDAFQLLLEGKCIRCVLWMG--

gi|3859714|emb|CAA21988.1|_alc AVDFFTRG------LIKCPIKIVGLSELPEVYKLMEEGKILGRYVLDNDK

gi|608690|emb|CAA57342.1|_alco AIDFFSRG------LIKCPIKIVGLSDLPEVFKLMEEGKILGRYVLDTSK

gi|171021|gb|AAA34408.1|_alcoh ALDFFARG------LVKSPIKVVGLSSLPEIYEKMEKGQIAGRYVVDTSK

gi|86278351|gb|ABC88428.1|_alc AIEFFRRG------LIKAPFKIVGLSELQMVYDKMHQGAVVGRYVLDTSK

gi|12643994|sp|P00332.2|ADH_SC ALEYVSRG------LVKPYYKVQPFSTLPDVYRLMHENKIAGRIVLDLSK

. : : : : : . :

The sequences of alcohol dehydrogenase (ADH) from various sources were aligned using Clustal W (1.81) multiple sequence alignment tool (<http://align.genome.jp/>) and manually optimized. The sequences are as follows: 1) Chain A of human alpha alcohol dehydrogenase, 2) Chain A, human beta-1 alcohol dehydrogenase, 3) Alcohol dehydrogenase E chain from *Equus caballus* (horse), 4) alcohol dehydrogenase from *Rattus norvegicus* (Norway rat), 5) Chain A of Mouse Alcohol Dehydrogenase from *Mus musculus*, 6) alcohol dehydrogenase 1 from *Zea mays*, 7) alcohol dehydrogenase from *Arabidopsis thaliana*, 8) alcohol dehydrogenase II from *Candida albicans*, 9) alcohol dehydrogenase from *Candida albicans*, 10) alcohol dehydrogenase II from *Saccharomyces cerevisiae*, 11) alcohol dehydrogenase from *Cochliobolus lunatus*, 12) alcohol dehydrogenase from [*Schizosaccharomyces pombe*](http://www.ncbi.nlm.nih.gov/Taxonomy/Browser/wwwtax.cgi?id=4896).

*Curvularia lunata* ADH (CADH) accession number is highlighted in blue whereas the conserved residues are denoted by an asterisk (*). CADH sequence shows the presence of all 22 residues conserved across kingdoms as reported by Jornvall et al.,1987 (40). These residues are highlighted in yellow.
